# BRD7 mediates hyperglycaemia‐induced myocardial apoptosis *via* endoplasmic reticulum stress signalling pathway

**DOI:** 10.1111/jcmm.13041

**Published:** 2016-12-13

**Authors:** Xiao‐meng Wang, Ying‐cui Wang, Xiang‐juan Liu, Qi Wang, Chun‐mei Zhang, Li‐ping Zhang, Hui Liu, Xin‐yu Zhang, Yang Mao, Zhi‐ming Ge

**Affiliations:** ^1^ Key Laboratory of Cardiovascular Remodeling and Function ResearchChinese Ministry of Education and Chinese Ministry of HealthQilu Hospital of Shandong UniversityJinanShandongChina; ^2^ Department of CardiologyQilu Hospital of Shandong University (Qingdao)QingdaoShandongChina; ^3^ Department of GeriatricsJinan Central Hospital Affiliated to Shandong UniversityJinanShandongChina

**Keywords:** BRD7, diabetic cardiomyopathy, endoplasmic reticulum stress, CHOP, apoptosis

## Abstract

Bromodomain‐containing protein 7 (BRD7) is a tumour suppressor that is known to regulate many pathological processes including cell growth, apoptosis and cell cycle. Endoplasmic reticulum (ER) stress‐induced apoptosis plays a key role in diabetic cardiomyopathy (DCM). However, the molecular mechanism of hyperglycaemia‐induced myocardial apoptosis is still unclear. We intended to determine the role of BRD7 in high glucose (HG)‐induced apoptosis of cardiomyocytes. *In vivo*, we established a type 1 diabetic rat model by injecting a high‐dose streptozotocin (STZ), and lentivirus‐mediated short hairpin RNA (shRNA) was used to inhibit BRD7 expression. Rats with DCM exhibited severe myocardial remodelling, fibrosis, left ventricular dysfunction and myocardial apoptosis. The expression of BRD7 was up‐regulated in the heart of diabetic rats, and inhibition of BRD7 had beneficial effects against diabetes‐induced heart damage. *In vitro*, H9c2 cardiomyoblasts was used to investigate the mechanism of BRD7 in HG‐induced apoptosis. Treating H9c2 cardiomyoblasts with HG elevated the level of BRD7 *via* activation of extracellular signal‐regulated kinase 1/2 (ERK1/2) and increased ER stress‐induced apoptosis by detecting spliced/active X‐box binding protein 1 (XBP‐1s) and C/EBP homologous protein (CHOP). Furthermore, down‐regulation of BRD7 attenuated HG‐induced expression of CHOP *via* inhibiting nuclear translocation of XBP‐1s without affecting the total expression of XBP‐1s. In conclusion, inhibition of BRD7 appeared to protect against hyperglycaemia‐induced cardiomyocyte apoptosis by inhibiting ER stress signalling pathway.

## Introduction

Diabetes is a primary cause of mortality and morbidity all over the world 1. More than half of diabetic patients are died of cardiovascular complications, including DCM, which results in changes of the structure and function in the heart independently of hypertension and underlying coronary artery disease 2. DCM is characterized by left ventricular hypertrophy and diastolic dysfunction 3. Diverse pathogenic mechanisms have been identified in DCM, including myocardial cell death, contractile protein glycosylation and interstitial fibrosis 4. In particular, myocardial cell apoptosis is a major component of DCM 5. Diabetic patients with dilated cardiomyopathy showed more apoptotic cardiomyocytes than patients without diabetes 6.

A previous study indicated that ER stress might contribute to the development of DCM, as the ERs became swollen under ultrastructural examination in the diabetic hearts 7. Whereafter, we have demonstrated that ER stress was involved in the cardiac apoptosis in STZ‐induced type 1 diabetic rat model 8. Chronic hyperglycaemia increases the level of ER stress, including glucose‐regulated protein 78 kD (GRP78), spliced/active XBP‐1s and CHOP 9. CHOP is identified to play a prominent role in ER stress‐induced apoptosis 10. CHOP can be induced at the transcript level by multiple pathways, such as XBP‐1s, activating transcription factor 4 and activating transcription factor 6 11. Although ER stress participates in the pathogenesis of DCM, the molecular mechanisms underlying cardiac apoptosis still have not been well illustrated.

BRD7 is a pleiotropic and highly conserved protein that is ubiquitously expressed in human tissues, including brain, heart, lung, colon and breast 12, 13. It is a member of the bromodomain‐containing protein family that is known as a tumour suppressor 14. BRD7 serves as a transcriptional regulation factor which regulates many pathological processes such as cell growth, apoptosis and cell cycle 15. Increasing evidence have demonstrated that BRD7 had distinct effects on inducing cell apoptosis. For example, BRD7 induced a significant increase in apoptosis of ovarian cancer cells in a p53‐independent manner 16. Liu *et al*. 17 has identified that BRD7 contributed to initiate apoptosis *via* repressing PTEN/AKT signalling in nasopharyngeal carcinoma. In addition, a recent study showed that BRD7 modulated ER stress through its ability to regulate XBP1s nuclear translocation in the liver of obese mice 18. Although many studies have examined the effects of BRD7, little is known about the function of BRD7 in DCM.

We have suggested that increased BRD7 expression may aggrandize diabetes or HG‐induced cardiomyocyte apoptosis, and BRD7 inhibition may have a protective effect on the myocardium in diabetes. Here, we investigated the potential role and underlying mechanism of BRD7 involved in HG‐induced cardiomyocyte apoptosis *in vivo* and *in vitro*.

## Materials and methods

### Animal models

Sixty male Wistar rats (mean body weight 200 ± 20 g) were obtained from Beijing Weitong Lihua Experimental Animal Technology (Beijing, China). The rats were housed at 22°C with an alternating 12 hrs light/dark cycle. After 1 week of acclimatization, the animals were then randomly divided into four groups (*n* = 15 each): normal control, diabetes mellitus (DM), DM+shRNA‐negative control (N.C) and DM+shRNA‐BRD7. Diabetic rats received a single intraperitoneally injection of STZ (60 mg/kg, Sigma‐Aldrich, St. Louis, MO, USA) dissolved in 0.1 ml of citrate buffer (pH 4.5) to induce diabetes. Normal rats were injected with citrate buffer only. One week after STZ injection, tail vein random glucose levels were measured using a glucometer (ACCU‐CHEK Advantage; Roche, Indianapolis, IN, USA). Rats with blood glucose levels >16.7 mmol/l were considered the type 1 diabetic rats. After induction of diabetes for 12 weeks, an amount of 1 × 10^8^ UT/50 μl of lentivector with BRD7 shRNA (GenePharma, Shanghai, China) or the same volume of lentivehicle (GenePharma) was injected into the jugular vein. At 16 weeks after STZ injection, all rats were anaesthetized with sodium amobarbital (35 mg/kg of body weight intraperitoneally) and then killed. All experiments conformed to the Guide for the Care and Use of Laboratory Animal published by the US National Institutes of Health and Shandong University. The study protocol was approved by the Institutional Ethics Committee of Shandong University. The target sequence for BRD7 shRNA was 5′‐GGACTCTGGAGATGCTGAA‐3′ and negative control sequence 5′‐TTCTCCGAACGTGTCACGT‐3′.

### Cardiac function measurement

Echocardiography was performed by use of the VEVO770 imaging system (VisualSonics, Toronto, ON, Canada) before lentivirus infection and at the end of the experiment. All rats were anaesthetized with sodium amobarbital (35 mg/kg of body weight intraperitoneally) to perform echocardiographic analysis. The left ventricular ejection fraction (LVEF), left ventricular fractional shortening (LVFS), left ventricular end‐diastolic diameter (LVEDd), left ventricular posterior wall thickness (LVPWd) and left ventricular mass (LV mass) were measured by M type ultrasound. A pulsed‐wave Doppler echocardiography was applied to determine the peak velocity of early (E) and late (A) ventricular filling velocity, and the ratio of early‐to‐late mitral inflow velocity (E/A) was calculated.

### Histology and immunohistochemistry

Rat hearts were dissected at the mid‐ventricular level and immediately fixed in 4% paraformaldehyde. Tissue samples were paraffin embedded and cut into 5‐μm sections for subsequent analyses. Cardiomyocyte width was manually determined as the shortest dimension per cardiomyocyte (μm), measured in images from haematoxylin and eosin (H&E)‐stained sections (×400 magnification within the left ventricle transverse sections) 19. Immunohistochemistry was performed for detecting the expressions of BRD7, and slides were incubated overnight at 4°C with the primary antibody rabbit anti‐BRD7 (Santa Cruz Biotechnology, Santa Cruz, CA, USA). Goat anti‐rabbit antibody was the secondary antibody. To detect interstitial collagen deposition, heart sections were stained with Masson's trichrome and Sirius red. All the results were analysed by use of IMAGE‐PRO PLUS 6.0.(Media Cybernatics, Houston, TX, USA)

### TUNEL staining

Apoptotic cells in myocardium were detected by use of a commercial DNA fragmentation detection kit (ApopTagPlus Peroxidase In Situ Apoptosis Detection Kit; Millipore, Billerica, MA, USA) according to the manufacturer's instructions. Briefly, H9c2 cardiomyoblasts were fixed with 4% paraformaldehyde for 15 min. at room temperature. Rat heart tissue sections were deparaffinized and hydrated. The samples underwent 20 μg/ml proteinase K for 5 min. and were washed with PBS. Then, samples were incubated with 3% H_2_O_2_ for 15 min. After adding the equilibration buffer, samples were incubated with TdT enzyme at 37°C for 1 hr. The samples were then incubated with antidigoxigenin conjugate at room temperature for 30 min. Peroxidase substrate was applied to detect apoptotic cells, stained brown and normal cells appeared green (0.5% methyl green‐pyronin).

### Cell culture

H9c2 cardiomyoblasts were incubated in six‐well plates in Dulbecco's modified Eagle's medium (DMEM) containing 10% foetal bovine serum (FBS) and 2 mM glutamine in 5% CO2 and 95% humidified air at 37°C. When cell populations reached 60% confluence, cells were exposed to normal glucose (NG; 5.5 mM glucose), high glucose (HG; 33.3 mM glucose) or high mannose [osmotic control (OC); 5.5 mmol/l glucose + 27.5 mmol/l mannose]. Cells were harvested at different times. Moreover, a specific ERK1/2 inhibitor (U0126; Selleck Chemicals, Houston, TX, USA) was used to identify the role of ERK1/2 in HG‐induced expression of BRD7. According to previous study 20, we chose 30 mM U0126 as the concentration in subsequent experiments. U0126 was added 30 min. before HG treatment, and dimethyl sulfoxide (DMSO; Sigma‐Aldrich) treatment was acted as negative control.

### BRD7 knockdown in H9c2 cardiomyoblasts

H9c2 cardiomyoblasts were cultured in six‐well culture plates and infected with a lentivirus vector containing BRD7‐shRNA at a multiplicity of infection 50 for 48 hrs, and then, cells were cultured with HG for 48 hrs. The target sequence for BRD7 shRNA was 5′‐GGACTCTGGAGATGCTGAA‐3′ and negative control sequence 5′‐TTCTCCGAACGTGTCACGT‐3′.

### Immunofluorescence microscopy

H9c2 cardiomyoblasts were cultured on glass coverslips in 2‐cm^2^ wells. For BRD7 and XBP‐1s analysis, cells were treated with HG for 48 hrs after using U0126 or transfection of shRNA BRD7. Immunofluorescence analysis was performed as described 21. Cells were incubated overnight at 4°C with primary antibodies for BRD7 (Santa Cruz Biotechnology) and XBP‐1s (Abcam, Cambridge, MA, USA) in PBS with 0.1% Triton X‐100 in a humidified chamber. Cells were washed with PBS and incubated with secondary antibody (1:200 dilution; Cell Signaling Technology, Beverly, MA, USA) for 30 min. at 37°C. Images were acquired by laser scanning confocal microscopy (LSM710; Zeiss, Jena, Germany).

### Western blot analysis

Total protein was collected from freshly dissected rat hearts and cell lysates. The nuclear and cytoplasmic proteins of H9c2 cardiomyoblasts were collected using a nuclear and cytoplasmic protein extraction kit (Beyotime Institute of Biotechnology, Jiangsu, China). Equal amounts of protein were separated on 10% or 12% sodium dodecyl sulphate–polyacrylamide gel electrophoresis (SDS‐PAGE) and then transferred to PVDF membranes (Millipore, Eschborn, Germany). The membranes were blocked for 2 hrs with 5% non‐fat milk at room temperature, then incubated overnight at 4°C with primary antibodies specifically against BRD7 (Sigma‐Aldrich), CHOP (Novus, Littleton, CO, USA), XBP‐1s (Abcam), cleaved caspase‐3, full length caspase‐3, B‐cell lymphoma/leukaemia‐2 (Bcl‐2), Bcl2‐associated X protein (Bax), β‐actin, GAPDH, histone3, phospho‐ERK1/2, total ERK1/2, phospho‐Akt and total‐AKT (all Cell Signaling Technology). After being washed three times, the membranes were incubated with respective secondary antibody for 90 min. at room temperature. Protein contents were visualized using an enhanced chemiluminescent reagent (Bio‐Rad, Hercules, CA, USA).

### Statistical analysis

All experiments were repeated at least three times. Data were presented as mean ± S.D. Differences between two groups were performed by unpaired *t*‐test, and multiple groups involved one‐way anova. Differences were considered statistically significant at *P* < 0.05. SPSS 17.0 (SPSS, Chicago, IL, USA) was used for statistical analysis.

## Results

### Diabetes increased myocardial BRD7 expression and BRD7 inhibition prevented diabetes‐induced myocardial remodelling and fibrosis *in vivo*

**Figure 1:**
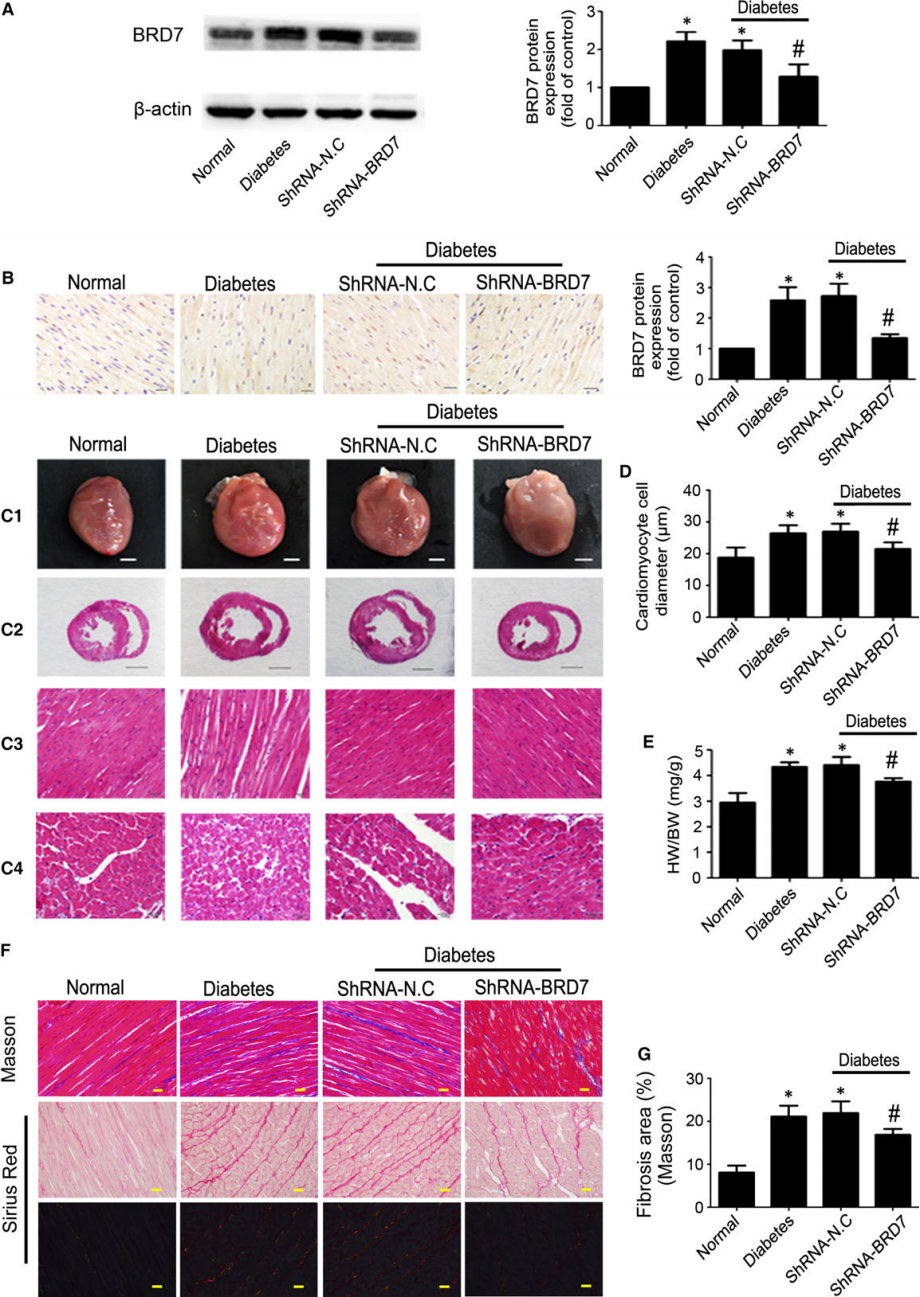
BRD7 expression and effects on myocardial pathology and fibrosis. (**A**) Western blot analysis of BRD7 protein levels. (**B**) Immunohistochemical staining and quantification of BRD7 expression (scale bar: 20 μm). (**C1**) Heart size (scale bar: 3 mm). (**C2**) Representative histologic cross‐sectional image illustrating the anatomy at the papillary muscle level (scale bar: 3 mm). (**C3**) Representative haematoxylin and eosin staining (HE) of longitudinal left ventricular (LV) sections (scale bar: 20 μm). (**C4**) Representative HE staining of LV transverse sections (scale bar: 20 μm). (**D**) Quantitative analysis of cardiomyocyte cell diameter. (**E**) Quantitative analysis of heart weight‐to‐body weight ratio (HW/BW). (**F**) Masson's trichrome staining (first row; scale bar: 20 μm) and picrosirius Red staining (second and third rows; scale bar: 20 μm) of myocardium. (**G**) Quantitative analysis of myocardial fibrosis. Data are mean ± S.D. *P < 0.05 *versus* normal; #*P* < 0.05 *versus* diabetes or diabetes + shRNA‐N.C.

Diabetic rats showed significantly increased BRD7 protein level in the heart as compared with normal controls, and the BRD7 expression was down‐regulated in shRNA BRD7 than in vehicle‐treated diabetic rats as indicated by Western blot (*P* < 0.05; Fig. 1A) and immunohistochemistry (*P* < 0.05; Fig. 1B). The increased cardiomyocyte width in diabetic rats was attenuated by BRD7 gene silencing (*P* < 0.05; Fig. 1C and D). The ratio of heart weight to body weight was significantly higher in diabetic than normal rats, and shRNA BRD7 treatment decreased the ratio of heart weight to body weight (*P* < 0.05; Fig. 1E) in diabetic rats.

Masson's trichrome and picrosirius red staining of heart sections showed severe myocardial fibrosis in diabetic rats. Quantitative analysis of Masson's trichrome staining revealed a significant increase of collagen deposition in diabetic as compared with controls, and shRNA‐BRD7 treatment reduced collagen deposition as compared with vehicle treatment (*P* < 0.05; Fig. 1F and G). In addition, the levels of blood glucose in the diabetic group were significantly higher at all time‐points than at baseline (*P* < 0.05; Table S1), with BRD7 gene silencing, the blood glucose levels did not altered compared with vehicle group (Table S1).

### BRD7 inhibition attenuated diabetes‐induced cardiac dysfunction

At 12 weeks after the induction of diabetes, the diabetic rats showed a moderate decrease in LVEF, LVFS and E/A compared with controls; LVEDd, LVPWd and LV mass were higher than controls (all *P* < 0.05; Table S2), and these data had no difference between three groups of diabetic rats (Table S2). At the end of experiment, compared with the normal control group, LVEF, LVFS and E/A were significantly lower, while LVEDd, LVPWd and LV mass were significantly higher in the diabetic rats (all *P* < 0.05; Table 1). Inhibition of BRD7 was associated with an improvement in LVEF, LVFS, E/A, LVEDd, LVPWd and LV mass compared with vehicle treatment (all *P* < 0.05; Table 1). Echocardiographic images of rat hearts were shown (Fig. S1).

**Table 1:** Comparison of echocardiographic parameters after inhibition of BRD7

	Heart rate (bpm)	LVEF (%)	LVFS (%)	LVEDd (mm)	LVPWd (mm)	LV mass (mg)	E/A
Normal	306.33 ± 41.199	74.41 ± 1.48	44.57 ± 1.13	6.47 ± 0.77	2.28 ± 0.17	1181.85 ± 153.96	1.48 ± 0.07
DM	288.00 ± 12.289	49.74 ± 7.13^a^	29.89 ± 1.99^a^	9.29 ± 0.43^a^	3.08 ± 0.24^a^	1639.22 ± 107.73^a^	0.68 ± 0.06^a^
DM+ShRNA‐N.C	294.67 ± 33.828	57.49 ± 3.52^a^	31.82 ± 2.52^a^	9.39 ± 0.26^a^	3.14 ± 0.34^a^	1654.06 ± 77.08^a^	0.69 ± 0.04^a^
DM+ShRNA‐BRD7	289.00 ± 7.211	69.99 ± 1.69^b^	39.15 ± 1.89^b^	7.83 ± 0.34^b^	2.65 ± 0.09^b^	1418.28 ± 43.46^b^	0.96 ± 0.09^b^

a *P* < 0.05 compared with the normal control group.

b *P* < 0.05 compared with the DM or DM+ShRNA‐N.C.

Data are expressed as mean ± S.D.

DM: diabetes mellitus; LVEF: left ventricular ejection fraction; LVFS: left ventricular shortening fraction; LVEDd: left ventricular end‐diastolic diameter; LVPWd: left ventricular posterior wall thickness; LV mass: left ventricular mass; E/A: early‐to‐late mitral flow.

### BRD7 inhibition alleviated ER stress‐induced myocardial apoptosis in diabetic rats

**Figure 2:**
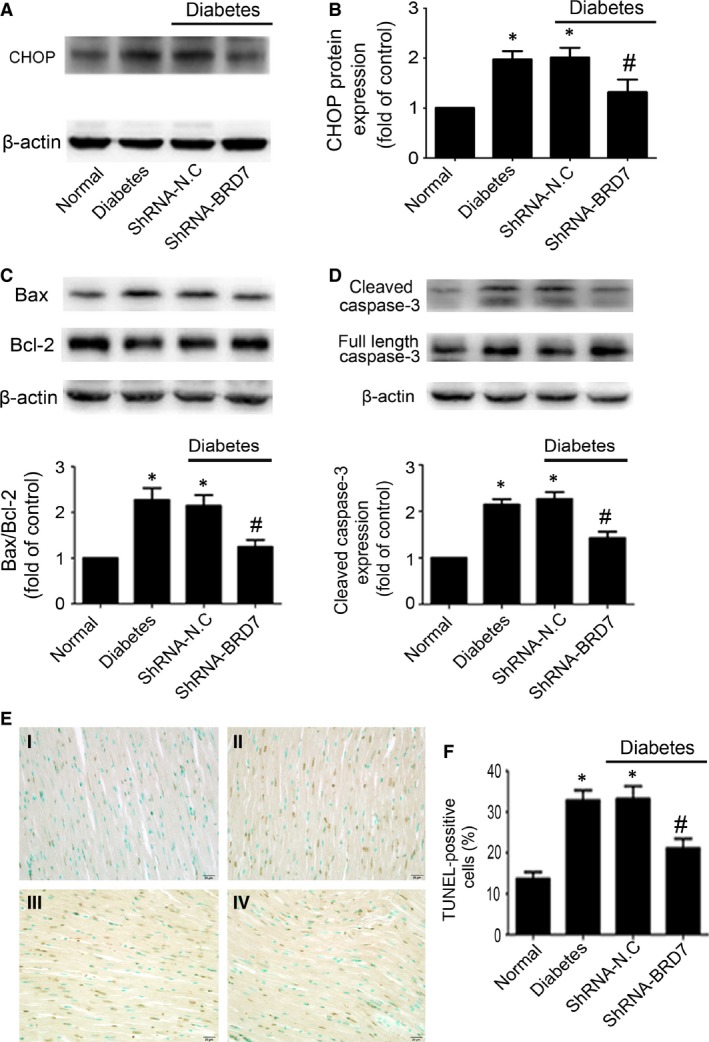
BRD7 inhibition protected against ER stress‐induced myocardial apoptosis in diabetic rats. (**A** and **B**) Western blot analysis of CHOP in diabetic rats. Bax and Bcl‐2 (**C**), the levels of cleaved caspase‐3 (**D**) after BRD7 inhibition were determined by Western blot. (**E** and **F**) TUNEL assay of cell apoptosis rate (scale bar: 20 μm). I: Normal; II: Diabetes; III: Diabetes+ shRNA‐N.C; IV: Diabetes+shRNA‐BRD7. Data are mean ± S.D. *P < 0.05 compared with normal; #*P* < 0.05 compared with diabetes or diabetes + shRNA‐N.C.

CHOP is a specific mediator of ER stress that is known to promote apoptosis in DCM 22. Diabetes significantly increased myocardial CHOP expression (*P* < 0.05; Fig. 2A and B), ratio of Bax/Bcl‐2 (*P* < 0.05; Fig. 2C) and caspase‐3 activity (*P* < 0.05; Fig. 2D). In addition, the proportion of TUNEL‐positive cells was significantly increased in diabetic hearts (*P* < 0.05; Fig. 2E and F). BRD7 inhibition effectively ameliorated diabetes‐induced CHOP expression (*P* < 0.05; Fig. 2A and B), ratio of Bax/Bcl‐2 (*P* < 0.05; Fig. 2C) and caspase‐3 activity (*P* < 0.05; Fig. 2D). Meanwhile, BRD7 inhibition decreased the proportion of TUNEL‐positive cells in the diabetic rats (*P* < 0.05; Fig. 2E and F). Thus, BRD7 was involved in ER stress‐induced myocardial apoptosis in diabetic rats.

### HG induced apoptosis of H9c2 cardiomyoblasts and increased the expression of BRD7

**Figure 3:**
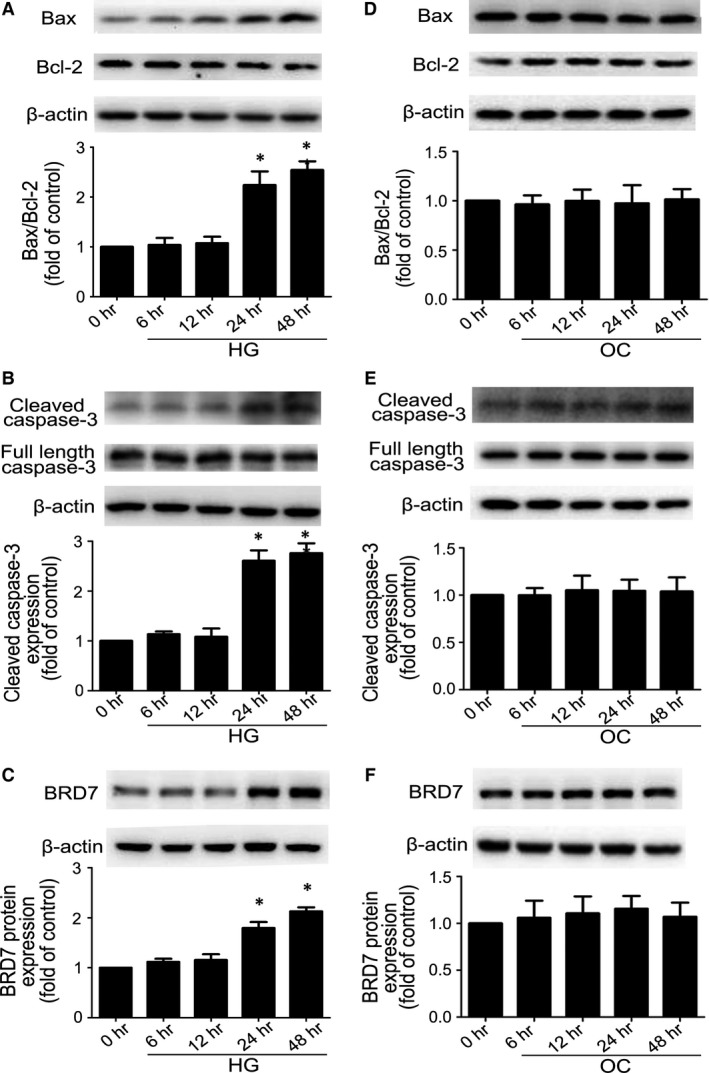
High glucose induced H9c2 cardiomyoblasts apoptosis and BRD7 expression. Bax and Bcl‐2 (**A**), the levels of cleaved caspase‐3 (**B**), the expression of BRD7 (**C**) with HG treatment was determined by Western blot. Bax and Bcl‐2 (**D**), the expression of cleaved caspase‐3 (**E**), the expression of BRD7 (**F**) with OC treatment was determined by Western blot. Quantitative data are expressed as fold of NG. Data are mean ± S.D. NG: normal glucose, 5.5 mmol/l glucose. HG: high glucose, 33 mmol/l. OC: osmotic control, 5.5 mmol/l glucose plus 27.5 mmol/l mannose. *P < 0.05 compared with time 0.

According to previous studies 23, 24, we chose an HG dose (33.3 mmol/l) to explore the effect of HG on apoptosis of H9c2 cardiomyoblasts, exposure of H9c2 cardiomyoblasts to HG for 6, 12, 24 and 48 hrs. The ratio of Bax to Bcl‐2 was increased with HG treatment at 24 and 48 hrs (both *P* < 0.05; Fig. 3A). The expression of cleaved caspase‐3 was higher with HG than NG (5.5 mmol/l) treatment at 24 and 48 hrs (both *P* < 0.05; Fig. 3B), while Bax/Bcl‐2 ratio and levels of cleaved caspase‐3 did not alter over time with OC as compared with NG (Fig. 3D and E). Meanwhile, we evaluated the BRD7 expression in HG‐treated H9C2 cardiomyoblasts at different times. The BRD7 protein level started to increase during the first 24 hrs and reached its peak at 48 hrs with HG (*P* < 0.05; Fig. 3C). Similarly, these effects were not observed with OC treatment (Fig. 3F). Forty‐eight hours was used as HG stimulation in subsequent experiments.

### BRD7 was required for HG‐induced H9c2 cardiomyoblasts apoptosis

**Figure 4:**
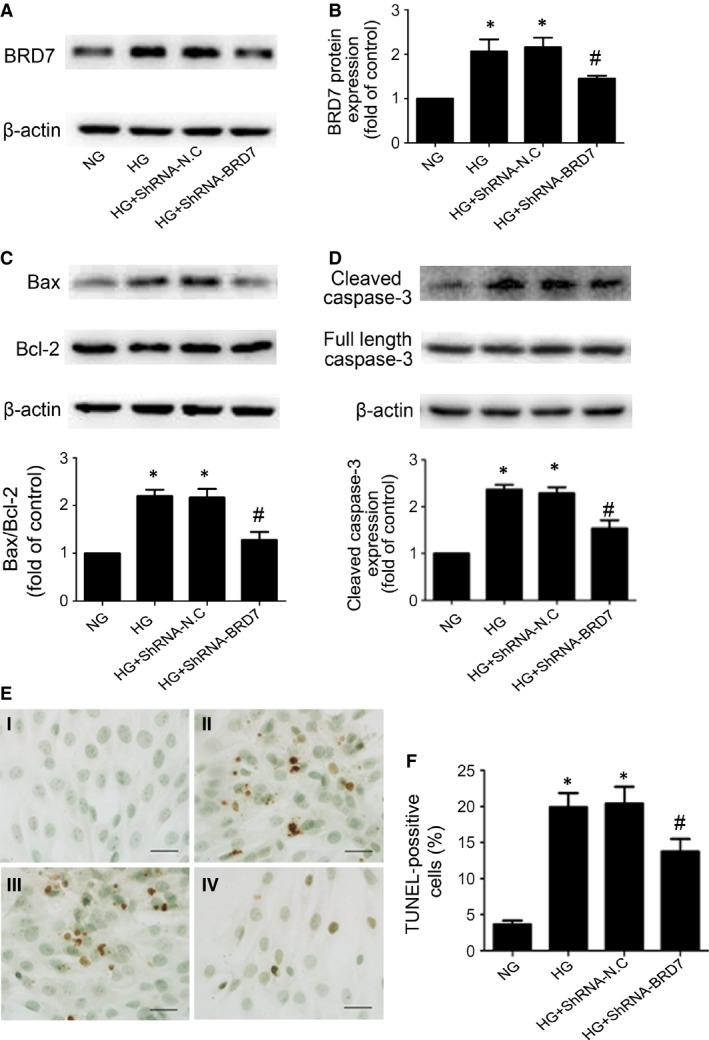
Inhibition of BRD7 reduced high glucose‐induced H9c2 cardiomyoblasts apoptosis. (**A** and **B**) Western blot analysis of the protein levels of BRD7 after transfected with BRD7‐shRNA or negative control shRNA (shRNA‐N.C). Western blot analysis of Bax and Bcl‐2 (**C**), the protein levels of cleaved caspase‐3 (**D**) with shRNA inhibition of BRD7. (**E** and **F**) TUNEL assay of apoptosis rate of H9c2 cardiomyoblasts (scale bar: 20 μm). I: NG; II: HG; III: HG+shRNA‐N.C; IV: HG+shRNA‐BRD7. Data are mean ± S.D. NG: normal glucose. HG: high glucose. *P < 0.05 compared with NG; #*P* < 0.05 compared with HG or HG + shRNA‐N.C.

To further study whether BRD7 was involved in HG‐induced H9c2 cardiomyoblasts apoptosis, we used BRD7‐specific shRNA to knockdown its protein level. The expression of BRD7 was significantly decreased after transfection with BRD7‐specific shRNA as compared with vehicle treatment (*P* < 0.05; Fig. 4A and B). HG stimulation significantly increased H9c2 cardiomyoblasts apoptosis, while inhibition of BRD7 attenuated Bax/Bcl‐2 ratio (*P* < 0.05; Fig. 4C), the level of cleaved caspase‐3 (*P* < 0.05; Fig. 4D) and as well as number of TUNEL‐positive cells (*P* < 0.05; Fig. 4E and F) induced by HG. These results demonstrated that HG‐induced H9c2 cardiomyoblasts apoptosis was reduced following the inhibition of BRD7.

### Inhibition of ERK pathway reduced HG‐induced expression of BRD7 and apoptosis

**Figure 5:**
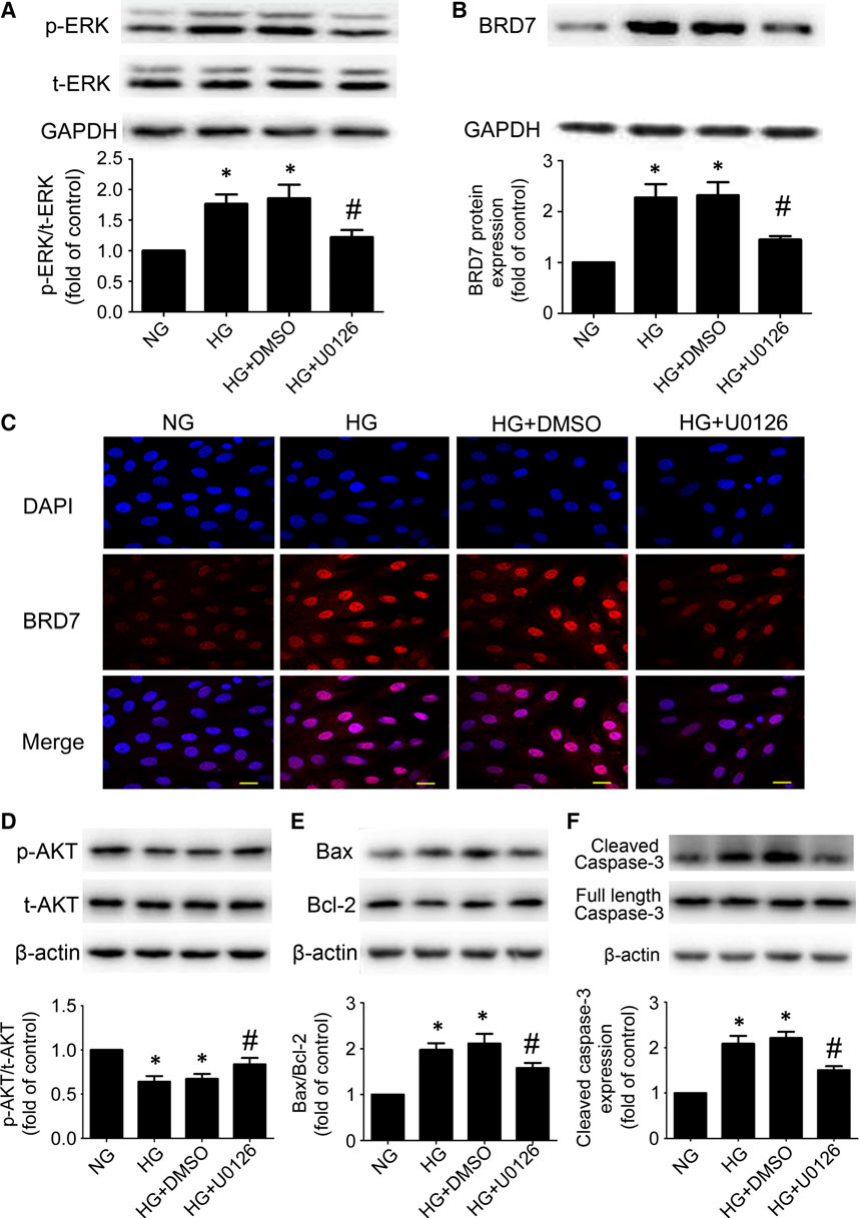
Inhibition of ERK pathway reduced high glucose‐induced expression of BRD7 and apoptosis. (**A**) Expression of total‐ERK (t‐ERK) and phospho‐ERK (p‐ERK) was measured by Western blot after using U0126. (**B**) Expression of BRD7 was measured by Western blot. (**C**) Confocal microscopy of BRD7 (scale bar: 20 μm). Western blot analysis of p‐AKT levels (**D**), Bax and Bcl‐2 (**E**), the protein levels of cleaved caspase‐3 (**F**) after using U0126. Data are mean ± S.D. NG: normal glucose. HG: high glucose. *P < 0.05 compared with NG; #*P* < 0.05 compared with HG or HG + DMSO.

Stimulation of H9c2 cardiomyoblasts with HG increased the phosphorylation of ERK1/2 level as compared with NG treatment (*P* < 0.05; Fig. 5A). To examine the role of ERK1/2 in HG‐induced expression of BRD7, we inhibited the activity of ERK1/2 using a specific ERK inhibitor (U0126). Pre‐treating H9c2 cardiomyoblasts with U0126 decreased phospho‐ERK1/2 level (*P* < 0.05; Fig. 5A) and the expression of BRD7 (*P* < 0.05; Fig. 5B) as compared with DMSO treatment. In addition, immunofluorescence analysis revealed that HG significantly increased the accumulation of BRD7 in the nucleus, which was attenuated by U0126 (Fig. 5C). We further explored the effects of ERK1/2 on HG‐induced apoptosis, HG stimulation markedly decreased AKT phosphorylation and p‐AKT level was enhanced by U0126 treatment (*P* < 0.05; Fig. 5D). Moreover, HG‐induced apoptosis was decreased in U0126 treatment group as indicated by Bax/Bcl‐2 ratio (*P* < 0.05; Fig. 5E) and the level of cleaved caspase‐3 (*P* < 0.05; Fig. 5F).

### BRD7 mediated HG‐induced apoptosis *via* ER stress pathway in H9c2 cardiomyoblasts

**Figure 6:**
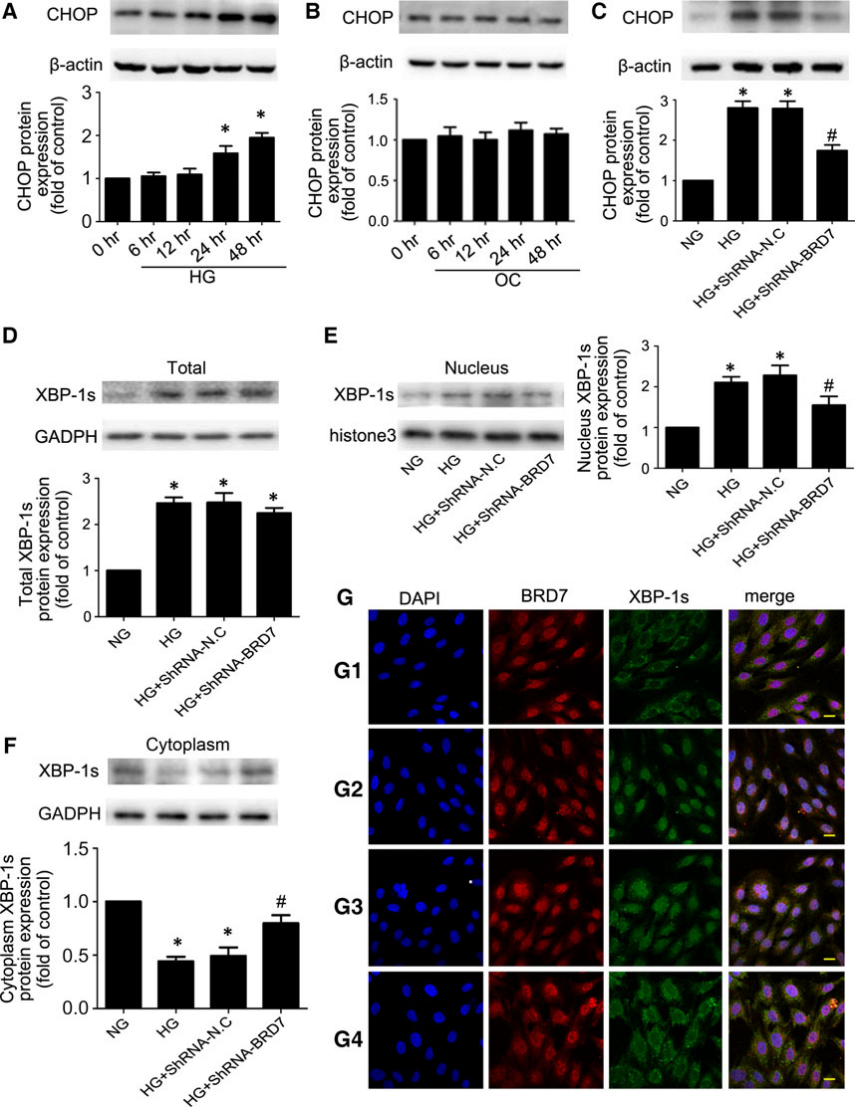
BRD7 mediated HG‐induced apoptosis *via* ER stress pathway in H9c2 cardiomyoblasts. (**A**) Expression of CHOP after HG stimulation for different times. (**B**) Expression of CHOP after OC stimulation for different times. (**C**) Expression of CHOP with shRNA inhibition of BRD7. Western blot analysis of the total expression of XBP‐1s (**D**), the nuclear expression of XBP‐1s (**E**) and the cytoplasmic expression of XBP‐1s (**F**) with shRNA inhibition of BRD7. (**G**) The localization of BRD7 and XBP‐1s was analysed by immunofluorescence (scale bar: 20 μm). BRD7 stained red; XBP‐1s stained green; nuclei stained blue with DAPI. Data are mean ± S.D. HG: high glucose. OC: osmotic control. *P < 0.05 compared with NG; #*P* < 0.05 compared with HG or HG + shRNA‐N.C.

To assess whether CHOP was associated with HG‐induced H9c2 cardiomyoblasts apoptosis, we explored CHOP expression for various times with HG treatment. After HG treatment for 24 hrs, the expression of CHOP was significantly increased and lasted to 48 hrs (*P* < 0.05; Fig. 6A). At the same time, the protein level of CHOP did not altered with OC treatment (Fig. 6B). In addition, Western blot showed that inhibition of BRD7 effectively decreased HG‐induced expression of CHOP (*P* < 0.05; Fig. 6C).

Then, we further investigated whether BRD7 promoted CHOP expression through XBP‐1s, Western blotting confirmed that HG increased the nuclear translocation of XBP‐1s, while its translocation was attenuated by inhibition of BRD7 (*P* < 0.05; Fig. 6E and F). Total expression of XBP‐1s was increased following exposure to HG as compared with NG (*P* < 0.05; Fig. 6D), and BRD7 inhibition had no effect on the total expression of XBP‐1s (Fig. 6D). Immunofluorescence analysis was also performed to monitor the localization of BRD7 and XBP‐1s. BRD7 was mainly located in the nucleus, and XBP‐1s was predominately in the cytosol of NG‐treated H9c2 cardiomyoblasts (Fig. 6G). After stimulation with HG, XBP‐1s was mainly located in the nucleus with coexpression of BRD7, while the nuclear XBP‐1s immunofluorescence signals were reduced upon BRD7‐shRNA treatment compared to vehicle treatment (Fig. 6G). Thus, BRD7 was involved in ER stress‐induced apoptosis of H9c2 cardiomyoblasts under HG treatment.

## Discussion

An increased myocardial cell apoptosis is a major event in the development of DCM 25. BRD7 has distinct effects on promoting cell apoptosis 26. However, its function in DCM is still unknown. Here, we focused on the potential role and mechanism of BRD7 in HG‐induced cardiomyocyte apoptosis. The major findings of our study were that *in vivo*, BRD7 protein levels were increased in the cardiomyocytes of type 1 diabetic rats, and inhibition of BRD7 prevented diabetes‐induced myocardial remodelling and fibrosis, improved cardiac dysfunction and limited myocardial apoptosis; *in vitro*, HG increased the expression of BRD7 by HG‐induced phosphorylation of ERK1/2; moreover, BRD7 mediated HG‐induced apoptosis through ER stress pathway. However, further detailed investigations are needed to clarify the exact molecular mechanism by which BRD7 modulates diabetic myocardial cell apoptosis *in vivo*.

Given that cardiomyocytes rarely proliferate, the apoptosis of cardiomyocytes would eventually result in compromised cardiac function and cardiac fibrosis 27, 28, 29. A previous study showed that in STZ‐induced diabetic rats, an increased cardiomyocyte apoptosis was observed 30. Meanwhile, HG significantly induced the apoptosis of H9c2 cardiomyoblasts 31. Consistent with previous studies, we found that the ratio of Bax/Bcl‐2 and the activity of caspase‐3 were enhanced in both diabetic hearts and HG‐stimulated H9c2 cardiomyoblasts. Additionally, the continuous loss of cardiomyocytes triggers myocyte hypertrophy, myocardial fibrosis and impaired systolic and diastolic function. Therefore, inhibition of cardiac apoptosis is an important strategy for the prevention of DCM.

BRD7 is a tumour suppressor and induces apoptosis in multiple cancers 32. However, the role of BRD7 in HG‐induced apoptosis of cardiomyocytes has not been characterized. In the present study, we found that BRD7 expression was increased in the type 1 diabetic rat heart and in HG‐treated H9c2 cardiomyoblasts. Moreover, BRD7 inhibition by shRNA reduced HG‐induced Bax/Bcl‐2 ratio and caspase‐3 activity both *in vivo* and *in vitro*. TUNEL assay also showed that the apoptosis of cardiomyocytes could be attenuated by inhibition of BRD7. In addition, we found that BRD7 inhibition ameliorated myocyte hypertrophy, myocardial fibrosis and cardiac dysfunction in DM rats. These results suggest that inhibition of BRD7 may protect cardiomyocytes against apoptosis under hyperglycaemic conditions.

Notably, it has been demonstrated that reinstating BRD7 levels in the liver improves insulin sensitivity to reduce the blood glucose levels in the obese and type 2 diabetic mouse 18. In our study, the blood glucose levels did not display a significant difference with BRD7 knockdown. Presumably, a high‐dose STZ caused rapid β cell destruction to inhibit insulin secretion 33. Although insulin resistance is present in type 1 diabetes, the severe insulin deficiency is the main contributor to hyperglycaemia in STZ diabetes 34.

We further investigated the potential mechanism of HG‐induced BRD7 expression *in vitro*. ERK1/2 is strongly activated by HG stimulation 35. A recent study showed that activation of Ras/Raf/MEK/ERK pathway increased BRD7 expression in hepatoma cell during HCV infection 36. Then, we investigated whether HG‐induced activation of ERK1/2 facilitated the expression of BRD7 in H9c2 cardiomyoblasts. In line with previous observation, we found that the phospho‐ERK1/2 level was significantly increased in HG‐stimulated H9c2 cardiomyoblasts, and pre‐treating cells with U0126 prevented HG‐induced phospho‐ERK1/2 level and expression of BRD7. These observations supported that HG mediated BRD7 expression *via* ERK1/2 pathway in H9c2 cardiomyoblasts. Interestingly, some studies have demonstrated that BRD7 down‐regulated the Ras/Raf/MEK/ERK pathway in several cancers 37, 38. Maybe there is a negative feedback mechanism between the activation of ERK1/2 and the expression of BRD7, and the balance between them is important to determine the outcome of many diseases. Further investigation is needed to explore the relationship in DCM. In addition, depending on the cell type and stimulus, ERK activation mediates various cell responses, such as proliferation, migration and death 39. Previous studies showed that activated ERK negatively controls the anti‐apoptotic AKT pathway 40. HMGB1 mediated hyperglycaemia‐induced cardiomyocyte apoptosis *via* ERK‐dependent activation of Ets‐1 23. Consistent with previous studies, we found inhibiting phosphorylation of ERK1/2 facilitated the activation of AKT, thus resulting in attenuated apoptosis in HG‐treated H9c2 cardiomyoblasts. These data confirmed that ERK pathway was involved in HG‐induced expression of BRD7 and apoptosis.

Increasing evidence suggest that ER stress participates in the apoptosis of DCM 41, 42. Our previous study has demonstrated CHOP promoted ER stress‐induced cardiomyocyte apoptosis by serving as a promotor of Puma in type 1 diabetic rats 43. In the present study, we found that cardiac CHOP level was significantly increased in diabetic rats, BRD7 inhibition reduced diabetes‐induced CHOP expression. *In vitro*, HG stimulation time‐dependently up‐regulated the expression of CHOP, and inhibiting BRD7 reduced the HG‐induced expression of CHOP. This suggests that increased BRD7 level promoted cardiac cell death *via* the transcription factor CHOP. In addition, a recent study showed that BRD7 forced XBP‐1s to the nucleus and increased the activity of XBP‐1s as a transcription factor in the liver of obese mouse 18. XBP‐1s is a highly active transcription factor and regulates ER folding capacity 44. It has shown that diabetes induced the expression of XBP‐1s in the mouse hearts 45. However, whether BRD7 mediated the activity of XBP‐1s in H9c2 cardiomyoblasts exposed to HG was not known. In our study, we found a marked up‐regulation of XBP‐1s in HG‐treated H9c2 cardiomyoblasts. Western blot and immunofluorescence analysis revealed that HG induced XBP‐1s to diffuse from the cytoplasm to the nucleus. Moreover, down‐regulation of BRD7 inhibited nuclear translocation of XBP‐1s, while the total expression was not altered. These observations indicated that BRD7 promoted nuclear translocation of XBP‐1s without affecting the total XBP‐1s protein levels. It is also believed that the CHOP promoter contains an ER stress response element motif which is known as an authentic XBP1‐bound sequence, and its transcription is modulated by XBP‐1s 46, 47. Thus, we speculated that BRD7 regulated the expression of CHOP *via* affecting the nuclear translocation and activity of XBP‐1s in HG‐treated H9c2 cardiomyoblasts. Interestingly, another study has demonstrated that XBP‐1s down‐regulated CHOP in chondrocytes and chondrosarcoma cells 48. Although the precise mechanisms for this difference are still unclear, it is possible that XBP‐1s regulates CHOP expression depending on the underlying pathological condition and cell context.

In conclusion, we illustrate that cardiac BRD7 gene silencing may protect against cardiac apoptosis and fibrosis, and improve myocardial function in diabetic rats. Additionally, BRD7‐ER stress signalling pathways play an essential role in hyperglycaemia‐induced cardiomyocyte apoptosis. However, the H9c2 cardiomyoblasts was derived from embryonic rat ventricular tissue, it has the feature of both skeletal and cardiac muscle cells 31. Although some studies have proved it has some similarities to primary cardiomyocytes and used as a substitution for cardiomyocytes, the extent to which H9c2 cardiomyoblasts can accurately simulate the responses of primary cardiomyocytes has not yet been fully established 49. Further investigations using primary cardiomyocytes should be needed to unravel these mechanisms.

## Author contributions

Xiao‐meng Wang designed the study, performed the research and wrote the manuscript. Ying‐cui Wang analysed the data. Xiang‐juan Liu wrote and edited the manuscript. Qi Wang, Chun‐mei Zhang, Li‐ping Zhang, Hui Liu, Xin‐yu Zhang and Yang Mao performed the research. Zhi‐ming Ge reviewed and edited the manuscript.

## Conflict of interests

The authors confirm that there are no conflict of interests.

## Supporting information


**Figure S1** Echocardiographic images of rat hearts.

**Table S1** Blood glucose (mmol/l) levels in rats in each experimental conditions.
**Table S2** Comparison of echocardiographic parameters at 12 weeks after STZ injection.
