# Supplementary figures and images for: BRD7 mediates hyperglycaemia‐induced myocardial apoptosis via endoplasmic reticulum stress signalling pathway
Source: J Cell Mol Med. 2016 Dec 13;21(6):1094–105. doi: 10.1111/jcmm.13041 (PMC5431142; doi:10.1111/jcmm.13041)

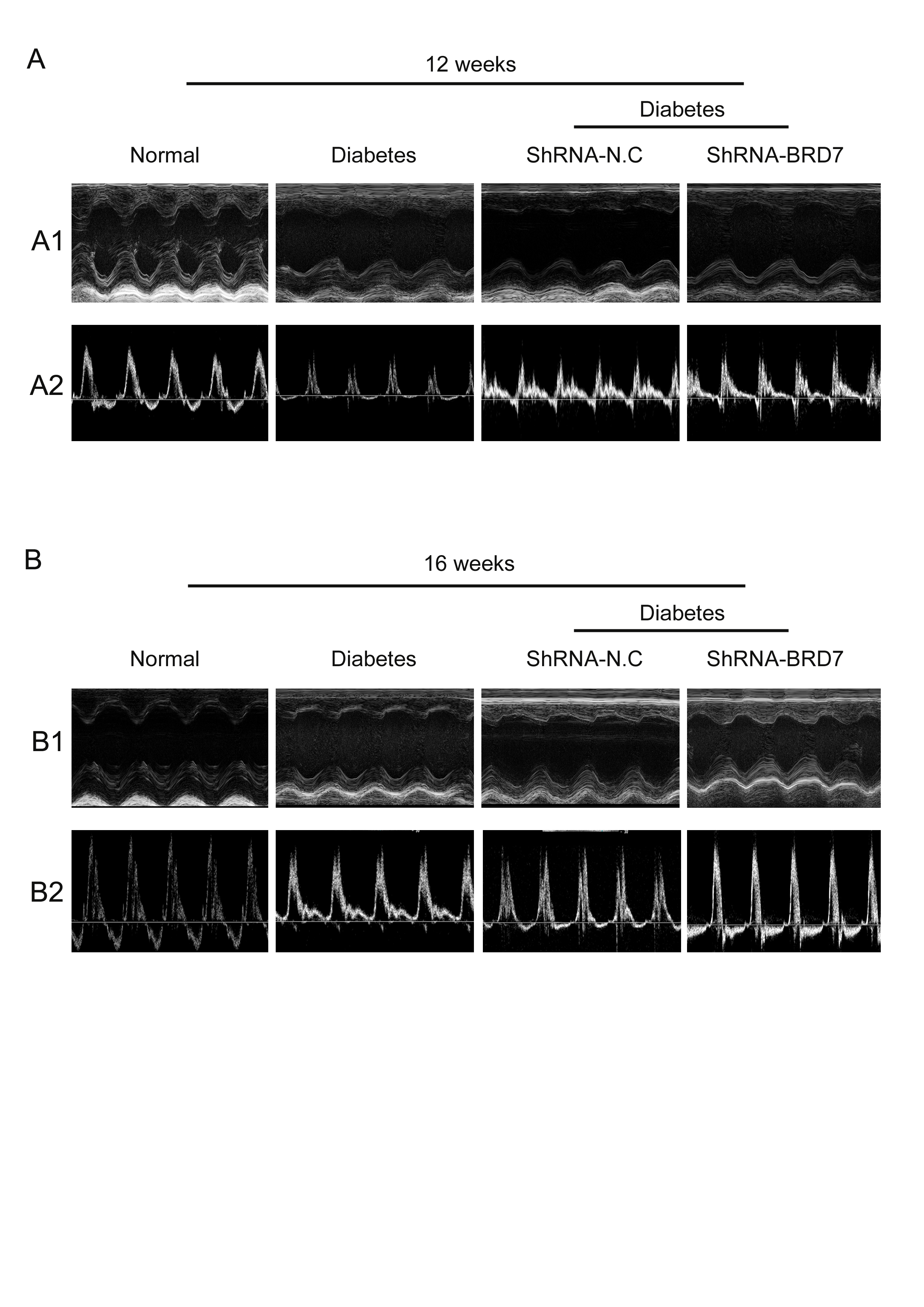

Supplement: Supplementary file 1 — Figure S1 Echocardiographic images of rat hearts. [file JCMM-21-1094-s001.tif]
