# Supplementary material for: BRD7 mediates hyperglycaemia‐induced myocardial apoptosis via endoplasmic reticulum stress signalling pathway
Source: J Cell Mol Med. 2016 Dec 13;21(6):1094–105. doi: 10.1111/jcmm.13041 (PMC5431142; doi:10.1111/jcmm.13041)
Supplement: Supplementary file 2 — Table S1 Blood glucose (mmol/l) levels in rats in each experimental conditions. Table S2 Comparison of echocardiographic parameters at 12 weeks after STZ injection. [file JCMM-21-1094-s002.docx]

**Supplementary table 1** Blood glucose (mmol/L) levels in rats in each experimental conditions

|  | baseline | 1 week | 12 weeks | 16 weeks |
| --- | --- | --- | --- | --- |
| Normal | 6.23 ± 0.763 | 6.70 ± 0.560 | 6.55 ± 1.439 | 6.53 ± 0.971 |
| DM | 7.05 ± 0.480 | 23.35 ± 1.515* | 25.20 ± 3.601* | 28.03 ± 1.307* |
| DM + ShRNA-N.C | 7.15 ± 0.742 | 24.23 ± 1.877* | 27.08 ± 3.041* | 28.50 ± 1.783* |
| DM + ShRNA-BRD7 | 7.05 ± 0.479 | 23.65 ± 2.495* | 26.88 ± 4.434* | 27.18 ± 2.955* |

*P < 0.05 compared with the normal control group. Data are expressed as mean ± SD. DM: diabetes mellitus.

**Supplementary table2** Comparison of echocardiographic parameters at 12 weeks after STZ injection

|  | Heart rate (bpm) | LVEF (%) | FS (%) | LVEDd (mm) | LVPWd (mm) | LV mass (mg) | E/A |
| --- | --- | --- | --- | --- | --- | --- | --- |
| Normal | 312.33±29.263 | 76.96 ± 2.29 | 43.88 ± 1.25 | 6.55 ± 0.45 | 2.06 ± 0.22 | 1057.55 ± 77.65 | 1.37 ±0.08 |
| DM | 280.67±16.503 | 55.10 ± 3.19* | 33.92 ± 1.98* | 8.89 ± 0.66* | 2.96 ± 0.33* | 1584.21 ± 99.77* | 0.75 ± 0.14* |
| DM+ShRNA-N.C | 297.33±17.616 | 58.84 ± 2.99* | 35.26 ± 3.33* | 8.95 ± 0.81* | 3.01 ±0.38* | 1600.71 ± 73.29* | 0.83 ± 0.11* |
| DM+ShRNA-BRD7 | 301.00±20.075 | 59.93 ± 4.25* | 35.03 ± 1.81* | 8.98 ± 0.55* | 3.03 ± 0.21* | 1552.65 ± 118.17* | 0.84 ± 0.25* |

*P < 0.05 compared with the normal control group.

Data are expressed as mean ± SD.

DM: diabetes mellitus; LVEF: left ventricular ejection fraction; FS: left ventricular shortening fraction; LVEDd: left ventricular end diastolic diameter; LVPWd: left ventricular posterior wall thickness; LV mass: left ventricular mass; E/A: Early to late mitral flow.
